# Instability in Pentanucleotide Markers in a Subset of Microsatellite Instability-High Colorectal Cancer

**DOI:** 10.3390/curroncol33040205

**Published:** 2026-04-02

**Authors:** Ahmet Yilmaz, Wendy L. Frankel, Benjamin J. Swanson, Kristin Miller, Jason Bacher, Christopher Bigley, Lori Nelsen, Matthew F. Kalady, Joshua F. Coleman, Rachel Pearlman, Heather Hampel

**Affiliations:** 1 Department of Pathology, The Ohio State University Wexner Medical Center, Columbus, OH 43210, USA; 2 Department of Pathology, Microbiology, and Immunology, The University of Nebraska Medical Center, Omaha, NE 68198, USA; 3 Division of Human Genetics, Department of Internal Medicine, The Ohio State University Wexner Medical Center, Columbus, OH 43210, USA; 4 Vanta Diagnostics, Charleston, SC 29403, USA; 5 Department of Surgery, The Ohio State University Wexner Medical Center, Columbus, OH 43210, USA; 6 Department of Pathology, University of Utah, Salt Lake City, UT 84112, USA; 7 Division of Clinical Cancer Genomics, Department of Medical Oncology and Therapeutics Research, City of Hope National Medical Center, Duarte, CA 91010, USA

**Keywords:** colorectal cancer, microsatellite instability, MSI, pentanucleotide, DNA mismatch repair, MMR, immunohistochemistry, IHC, short tandem repeats, STR

## Abstract

Microsatellite instability (MSI) testing is frequently used to screen patients for the early detection of Lynch syndrome, the most common hereditary colorectal cancer syndrome. MSI testing compares microsatellite repeat lengths in tumor DNA with those in matched normal tissue from the same patient. Therefore, precise sample identification is critical for obtaining reliable test results. The Penta-C and Penta-D pentanucleotide markers are widely used for sample identification in MSI testing. We investigated instability, defined as allelic mismatches or shifts, discordant fragment sizes, or the appearance of alleles in tumor DNA that were absent in the corresponding normal DNA, in the Penta-C and Penta-D loci across 2609 paired colorectal tumor and matched normal tissue or blood DNA samples. The allele sizes of both markers did not match in 0.3% of microsatellite-stable (MSS) and 12.3% of microsatellite instability-high (MSI-H) patients (*p* < 0.001, difference in proportions, 12.0% (95% CI, 8.9–15.1%)). Non-matching allele sizes in 12.3% of the MSI-H tumors suggest that other repeat markers may also be unstable and not suitable for sample identification in these tumors.

## Simple Summary

Colorectal cancer (CRC) is the second leading cause of cancer-related deaths in the U.S. Microsatellite instability (MSI) testing is frequently used to screen patients for Lynch syndrome, also known as hereditary nonpolyposis colorectal cancer (HNPCC), the most common hereditary CRC syndrome. We investigated instability in the Penta-C and Penta-D pentanucleotide markers used for sample identification in MSI testing in 2609 pairs of normal tissue (or blood) and matched tumor DNA samples from patients with CRC. Allele sizes for both markers did not match in 0.3% of microsatellite-stable (MSS) and 12.3% of microsatellite instability-high (MSI-H) tumors (*p* < 0.001). Therefore, other repeat loci may also be unstable, and additional identification methods may be necessary to ensure accurate sample matching in MSI-H CRC patients.

## 1. Introduction

Microsatellites are short (1–6 bp) tandem repeats of nucleotides dispersed throughout the genome [1,2]. The repetitive nature of microsatellites promotes DNA strand slippage during replication. Although replication errors that alter the number of repeat nucleotides are common in these regions, they are precisely repaired by DNA mismatch repair (MMR) proteins including MLH1, PMS2, MSH2, and MSH6 [3,4]. Defective MMR (dMMR), resulting from mutations in the genes encoding these proteins or from promoter hypermethylation, as in sporadic cases, leads to unrepaired changes in allelic size, termed microsatellite instability (MSI) [5,6].

Colorectal cancer (CRC) is the second leading cause of cancer-related deaths in the United States. Early detection is one of the strongest predictors of survival. The five-year survival rate is approximately 92% in early-stage disease but only 12% in late-stage disease [7]. Microsatellite instability testing is frequently used to screen patients for Lynch syndrome (LS) because over 90% of CRCs from patients with LS, the most common hereditary CRC syndrome, are microsatellite-unstable [1]. Currently, MSI testing is recommended as part of universal screening for all individuals with CRC because both LS patients and their relatives benefit substantially from early detection [8,9]. However, LS screening protocols have limitations in identifying individuals with LS [10]. Therefore, new methods or improvements to existing methodologies are needed to achieve more effective LS screening.

Microsatellite instability testing is also used to identify CRC patients who are candidates for immunotherapy. Multiple studies have shown that the presence of MSI is associated with improved response rates to immune checkpoint blockade therapy. The checkpoint inhibitors nivolumab and ipilimumab have demonstrated significant benefits in patients with MSI-H cancers, including improvements in quality of life, functional status, and symptom control, even among those who had failed prior treatments [11,12,13]. Consistently, a recent meta-analysis reported favorable clinical outcomes with checkpoint inhibitors, showing improved overall and progression-free survival in 939 patients across 14 studies involving various cancer types [14]. Patients in all but two of these studies had previously received at least one line of therapy.

Several diagnostic systems have been developed to detect MSI [15]. The Promega MSI Analysis System™, which includes the mononucleotide markers BAT-25, BAT-26, MONO-27, NR-21, and NR-24, has been widely used for MSI detection [1,10,16,17,18]. In this system, the allele sizes of these mononucleotide repeat markers in normal and tumor DNA from the same patient are compared. Samples showing no instability are classified as microsatellite stable (MSS); those with instability in one marker are classified as microsatellite instability-low (MSI-low); and those with instability in two or more markers are classified as microsatellite instability-high (MSI-H). The system also includes the pentanucleotide markers Penta-C and Penta-D, which are used to detect contamination or sample mix-ups. These markers are also incorporated into many commercial short tandem repeat (STR)-based identification kits developed for various purposes [19]. Penta-D, often utilized to study human population genetics and migration patterns [20], is among the most polymorphic STR markers known [21,22].

Despite their widespread use, data on possible instability, defined as allelic mismatches or shifts, discordant fragment sizes, or the appearance of alleles in tumor DNA that were absent in the corresponding normal DNA, in Penta-C and Penta-D are very limited in the literature. We recently reported pentanucleotide instability in patients with MSI-H endometrial cancer (EC) [23]. The objective of the present study was to investigate instability in these markers in CRC patients enrolled in the Ohio Colorectal Cancer Prevention Initiative (OCCPI) using the Promega Microsatellite Instability Analysis System™ v1.2 (Promega Inc., Madison, WI, USA). In addition, we examined correlations between pentanucleotide instability, clinical parameters, and electropherogram peak profiles to further assess the role of dMMR in instability at these loci.

## 2. Materials and Methods

### 2.1. Patients

In total, 3310 unselected patients with CRC were accrued from 51 hospitals across Ohio and enrolled in OCCPI, a statewide study designed to screen all CRC patients in Ohio for dMMR using MSI testing and/or immunohistochemistry (IHC) for the four MMR proteins MLH1, MSH2, MSH6, and PMS2 [24]. Tumors that were MSI-H and/or showed loss of MLH1/PMS2 expression by IHC underwent additional testing for hypermethylation of the *MLH1* promoter. Of the 3310 patients, 2609 were tested at The Ohio State University Wexner Medical Center and had available data for the Penta-C and Penta-D markers. The investigators had access to information that could identify individual participants during and after data collection. Testing was performed in a CLIA-certified laboratory, and results were provided to participants and their enrolling clinicians. All participants and/or their legal guardians provided written informed consent. Institutional Review Board (IRB) approval was obtained from participating hospitals, community oncology programs, or through reliance agreements with The Ohio State University IRB. No minors were included in the study. The methods have been described previously [24] and are summarized below.

### 2.2. Microsatellite Instability Assay

DNA was extracted from formalin-fixed, paraffin-embedded (FFPE) tissue sections containing at least 30% viable tumor cells. DNA isolation was performed using either the QIAamp DNA Mini Kit (Qiagen, Germantown, MD, USA) or the KingFisher automated DNA extraction system (Thermo Fisher Scientific, Waltham, MA, USA). Microsatellite instability testing was conducted using the Promega MSI Analysis System™ v1.2 (Promega Corporation, Madison, WI, USA). A two-step multiplex polymerase chain reaction (PCR) was carried out to amplify the designated markers using a GeneAmp 9700 PCR System thermocycler (Applied Biosystems, Carlsbad, CA, USA). Amplified products were separated by fluorescent fragment analysis via capillary electrophoresis on ABI 310 or 3130 Genetic Analyzers (Applied Biosystems). Electropherograms were analyzed using GeneMapper Software v5 (Applied Biosystems).

### 2.3. Determination of Electropherogram Peak Patterns in Unstable Mono- and Pentanucleotide Markers

**Figure 1:**
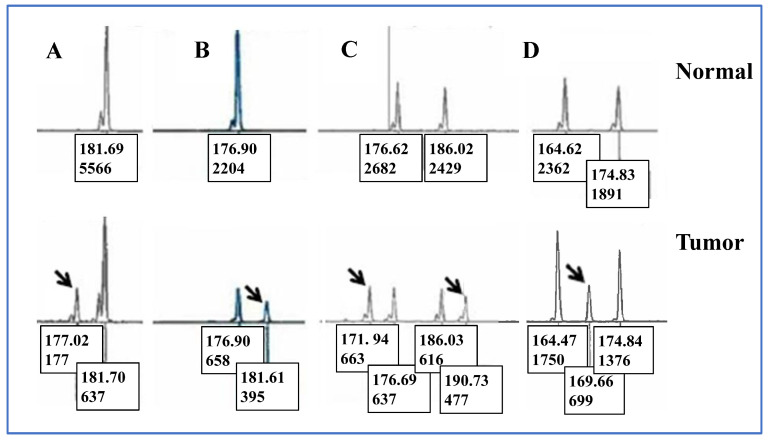
Representative examples of pentanucleotide repeat instability. (**A**) Deletion in a tumor sample resulting in a new allele approximately 5 bp shorter than the corresponding germline allele. (**B**) A duplication-type event producing a new allele approximately 5 bp longer than the germline allele. (**C**) Two possibly distinct instability events in the same sample generating two novel alleles: the shorter (171.94 bp) representing a deletion and the longer (190.73 bp) representing a duplication. (**D**) A new 169.66 bp allele observed in the tumor sample suggests instability; however, it cannot be determined whether this allele resulted from duplication in the 164.47 bp allele or deletion in the 174.84 bp allele. Such cases were classified as “indeterminate”. Figure is not drawn to scale.

We categorized the electropherogram peak patterns observed in unstable Penta-C and Penta-D loci into the following groups (Figure 1): “5 bp DEL” (a 5 bp deletion in a single peak), “5 bp DUP” (a 5 bp duplication in a single peak), “5 bp DEL short” (a 5 bp deletion occurring in the shortest of two or more peaks), and “5 bp DUP tall” (a 5 bp duplication occurring in the tallest of two or more peaks). Samples were assigned to the “indeterminate” category if a novel allele appeared between germline alleles such that it could not be determined whether the change resulted from deletion or duplication event(s). Samples displaying multiple alterations were classified as “complex”.

**Figure 2:**
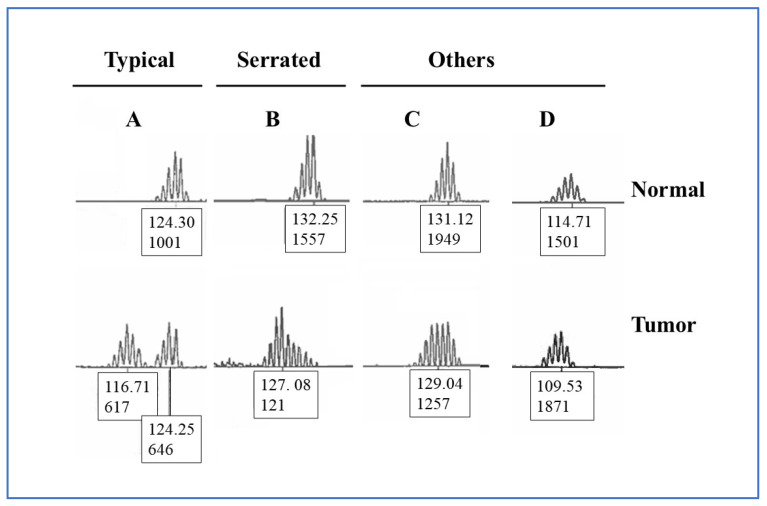
Representative examples of mononucleotide repeat instability. (**A**) Typical: two distinct peaks of different fragment sizes. (**B**) Serrated: multiple peaks with incremental variation in fragment height. (**C**,**D**) Others: peak patterns not classified as typical or serrated. This group includes (**C**) “flat” profiles, characterized by peaks of similar height clustered near the center, producing a flattened appearance, and (**D**) left-shifted profiles showing a (near-total) absence of normal tissue-specific peaks on the electropherogram. Figure is not drawn to scale.

Peak patterns in the unstable mononucleotide repeat markers BAT-25, BAT-26, MONO-27, NR-21, and NR-24 were categorized into three groups based on peak morphology and position on the electropherogram (Figure 2): “Typical” (two distinct peaks of different fragment sizes), “Serrated” (multiple peaks showing incremental variation in fragment height), and “Others” which included patterns such as a complete leftward shift on the electropherogram due to a near-total absence of normal-specific peaks, and “flat” profiles characterized by peaks of similar height clustered near the center, producing a flattened appearance. Differences of one bp or less between tumor and matched normal samples were ignored because such minimal size variation may reflect assay run variation rather than true instability.

### 2.4. Immunohistochemistry

Immunohistochemistry was performed using the two-stain method as previously described [24]. Staining for all four MMR proteins, MLH1, MSH2, MSH6, and PMS2, was carried out in cases with inconsistent or indeterminate MSI results. The antibodies used for IHC were obtained from Abcam (Waltham, MA, USA), Cell Signaling Technology (Danvers, MA, USA), Biocare Medical (Pacheco, CA, USA), and Vector Laboratories (Newark, CA, USA). Detailed IHC protocols have been published previously [24].

### 2.5. MLH1 Hypermethylation Assay

Five hundred nanograms of genomic DNA was bisulfite-converted using the EZ DNA Methylation-Gold™ Kit (Zymo Research, Irvine, CA, USA). *MLH1* promoter hypermethylation was assessed using the PyroMark Q96 *MLH1* Methylation Assay on a PyroMark Q96 ID pyrosequencer (Qiagen). Each run included fully methylated (SW48) and unmethylated (SW480) control DNA, as well as 5% and 50% SW48 DNA diluted in SW480 DNA. Percent methylation at the four CpG sites was averaged to determine the overall methylation status of each sample following manufacturer’s protocols as described previously [25].

### 2.6. Statistical Analysis

Statistical analyses were performed using t-test for continuous and Chi-square or Fisher’s exact test for categorical variables with MedCalc v20 (MedCalc Software Ltd., Ostend, Belgium) or GraphPad Prism v5.03 (GraphPad Software, San Diego, CA, USA); when investigating associations of peak patterns in unstable mononucleotide repeat markers with clinical parameters, *p* values were adjusted to control the family-wise error rate for multiple comparisons as described previously [26,27,28]. Holm critical value was computed using the formula

$${\alpha^{\prime }}_{i}=\frac{\alpha }{m-\mathrm{rank}\left(p_{i}\right)+1}$$

where ${\alpha^{\prime }}_{i}$ is the Holm-adjusted significance threshold for the *i*-th *p*; α represents the overall familywise error rate set at 0.05; m represents the total number of hypotheses being tested; and $\mathrm{rank}\left(p_{i}\right)$ is the rank of $p_{i}$ in ascending order such that smallest *p* receives a rank of “1”. $H_{\left(i\right)}$ was rejected when $p_{\left(i\right)}<{\alpha^{\prime }}_{i}$. Testing was stopped at the first instance of a non-significant result where the null hypothesis was not rejected [23,27].

## 3. Results

### 3.1. Clinical Characteristics of Patients

Table 1 summarizes the clinical characteristics of the 2609 patients with valid Penta-C and Penta-D data. Among all patients, 20.6% were younger than 50 years at diagnosis. Approximately 7.9% of patients were Black, 1.0% were Asian, and nearly all the remaining patients were White. Tissue samples were derived from the colon in 74.6% of cases, from the rectum in 23.1%, and from metastatic or other sites (e.g., liver, omentum) in the remainder. Overall, 440 patients (16.9%) were classified as MSI-H, and 83.5% (259/310) of tumors with loss of MLH1/PMS2 expression showed *MLH1* promoter hypermethylation. All four MMR proteins were intact in 82.9% (2158/2603) of patients. Loss of MLH1/PMS2 was observed in 12.4% (322/2603), loss of MSH2/MSH6 in 1.9% (49/2603), isolated loss of PMS2 in 1.1% (28/2603), and isolated loss of MSH6 in 0.8% (20/2603).

### 3.2. Instability in Penta-C and Penta-D Is More Frequent in MSI-H than MSS Samples

Table 2 summarizes the percentage of allelic size concordance between normal and tumor DNA in the Penta-C and Penta-D loci from the same patients. When analyzed separately, Penta-C and Penta-D did not match in 27.0% and 39.8% of MSI-H cases and in 2.1% and 2.0% of MSS cases, respectively. Risk ratios for Penta-C and Penta-D mismatch in MSI-H versus MSS were 12.7 (95% Confidence Interval (CI), 9.2–17.6) and 19.98 (95% CI, 14.6–27.5), respectively. When both pentanucleotide loci from the same patient were considered together, 12.3% of MSI-H and 0.3% of MSS cases showed allelic size mismatch (*p* < 0.001, difference in proportions, 12.0% (95% CI, 8.9–15.1%)). Risk ratio for mismatches in both Penta-C and Penta-D in MSI-H versus MSS, excluding partial mismatches, was 73.8 (95% CI, 32.1–169.9), suggesting that MSI status was significantly associated with allelic discordance when both pentanucleotide markers were considered.

Normal (germline) DNA was obtained from three sources depending on sample availability: (a) normal tissue on the same block as the tumor, (b) normal tissue on a separate block, or (c) peripheral blood when no normal tissue was available. The source of normal DNA was not significantly associated with instability in Penta-D (*p* = 0.65). However, there was a trend toward lower Penta-C mismatch rates in DNA isolated from blood (4.5%) compared with normal tissue blocks with (6.8%) or without (8.4%) tumor (*p* = 0.07).

### 3.3. Associations of Instability in Penta-C and Penta-D with Clinical Parameters

Patients were grouped according to Penta-C and Penta-D matching status, and data on *MLH1* promoter hypermethylation, age younger than 50 years at the time of diagnosis, MMR protein expression by IHC, and race were compared among groups (Table 3). The presence of *MLH1* promoter hypermethylation was associated with greater instability in Penta-C (*p* = 0.03). Loss of MLH1/PMS2 expression by IHC was significantly associated with instability in both Penta-C and Penta-D (*p* < 0.01), suggesting that the observed instability was likely attributable to dMMR. Unstable mononucleotide markers were more frequent in samples exhibiting instability in Penta-C (3.56 vs. 0.65) and Penta-D (3.95 vs. 0.59) than in samples without such instability.

### 3.4. Peak Patterns in Unstable Penta-C and Penta-D Are Not Significantly Different

Table 4 summarizes peak pattern distributions in unstable pentanucleotide markers. The proportions of individual peak patterns did not differ significantly between Penta-C and Penta-D (*p* > 0.40). A 5 bp deletion in the shortest of two or more peaks (“5 bp DEL short”) was the most frequent type of instability in both Penta-C and Penta-D, accounting for 21.5% and 25.7% of all mismatched cases, respectively.

### 3.5. Associations of Peak Patterns in Unstable Penta-C and Penta-D with Clinical Parameters

Patients were grouped according to peak pattern types in unstable Penta-C and Penta-D, and clinical parameters were compared among groups (Table 5). The “5 bp DUP tall” peak type in Penta-D was significantly more frequent in samples from patients younger than 50 years at diagnosis compared with those aged ≥ 50 years (40.5% vs. 3.6%; *p* < 0.01).

### 3.6. Penta-C Is More Stable than Penta-D in MSI-H Samples

Supplementary Table S1 summarizes the total number of peaks detected in Penta-C and Penta-D loci. In MSS samples, double peaks were present in approximately 81% of Penta-D and 74% of Penta-C electropherograms, with nearly all remaining samples displaying single peaks. Among MSI-H tumors, triple peaks were 10.7% more frequent in Penta-D than in Penta-C (27.7% vs. 17.0%), suggesting greater instability in Penta-D compared with Penta-C.

### 3.7. Associations Between Peak Pattern Types in Mononucleotide Repeat Markers and Instability in Penta-C and Penta-D Loci

To further verify the potential role of dMMR, assessed by instability in mononucleotide repeat markers, in pentanucleotide instability, patients were grouped according to peak pattern types in the mononucleotide markers, and Penta-C and Penta-D instability was evaluated in each group (Supplementary Table S2). Interestingly, “serrated” BAT-25 peaks were significantly more frequent in samples with matching than in those with non-matching Penta-C profiles (93.9% vs. 6.1%; *p* < 0.01). In addition, serrated peaks in MONO-27 were more frequent in samples with matching than non-matching Penta-D (79.5% vs. 20.5%; *p* < 0.01).

### 3.8. Associations Between Peak Pattern Types in Mononucleotide Repeat Markers and Clinical Parameters

Prompted by the observed associations between pentanucleotide peak patterns and both clinical parameters and mononucleotide peak patterns, patients were grouped according to peak pattern types in the mononucleotide repeat markers, and clinical characteristics were compared among groups (Supplementary Table S3). *MLH1* promoter hypermethylation, but not age at diagnosis, was associated with peak pattern types in NR-24 and BAT-25 (*p* < 0.01). Race was not associated with peak patterns (Supplementary Table S4). Analysis by race included only Black and White patients due to the small sample sizes in other groups.

## 4. Discussion

In this study, we investigated the nature and extent of instability in the Penta-C and Penta-D pentanucleotide repeat markers across 5218 tumor and matched normal tissue or blood DNA samples obtained from 2609 patients enrolled in the OCCPI clinical trial. Both pentanucleotide markers did not match in 12.3% of MSI-H and 0.3% of MSS cases. The presence of instability in both mono- and pentanucleotide markers in the MSI-H patients suggests a generalized form of genomic instability, indicating that additional nucleotide repeat loci may also be affected and, therefore, not suitable for sample identification in MSI-H tumors. These findings may help guide the design of improved MSI analysis systems in the future. Incorporating supplemental identification markers may enhance the reliability of sample matching in MSI-H patients exhibiting instability in pentanucleotide markers (Table 2).

Instability in pentanucleotide markers observed in MSI-H samples in our study is most likely attributable to dMMR, rather than to sample mix-ups or contamination. This conclusion is supported by the finding that high allele discordance occurred almost exclusively in MSI-H, but not in MSS samples despite all specimens being processed concurrently in randomized batches. The original alleles were retained in the tumors, and the novel peaks detected in tumor DNA were almost always in multiples of 5 bp, consistent with true instability events. Furthermore, MSI results were confirmed by IHC performed in the same laboratory using FFPE tissue sections cut from the same blocks used for MSI testing. A subset of samples underwent confirmatory analysis by ColoSeq^®^, which demonstrated greater than 99% concordance with the MSI results.

The 0.3% discordance observed among MSS patients is within the reported acceptable rate of sample misidentification (<0.5%) and the 0.2–0.6% discordance range reported by the developer of the MSI Analysis System [10]. This level of mismatch likely reflects either true sample mix-ups or the inherent limit of discrimination between individuals based on Penta-C and Penta-D loci. In contrast, it is not possible to determine an equivalent rate for MSI-H samples due to additional discordance arising from instability within the pentanucleotide markers.

Mismatches in pentanucleotide allele size were analyzed in relation to clinical parameters (Table 3). Penta-C mismatches were significantly more frequent in patients with *MLH1* promoter hypermethylation than in those without (*p* = 0.03). The absence of MMR protein expression was also associated with increased mismatches in both Penta-C and Penta-D (*p* < 0.01; Table 3). Notably, the pentanucleotide markers matched in all 37 MSI-H samples that were IHC-intact or showed isolated loss of MSH6. Although the sample size was small, reflecting the rarity of IHC-intact MSI-H tumors, this finding may be important for future studies, as it suggests that pentanucleotide loci remain stable when at least three of the four core MMR proteins (MLH1, PMS2, and MSH2) are expressed. The isolated loss of MSH6, a component of the MutSα complex responsible for mismatch recognition [29], appears insufficient to destabilize pentanucleotide repeats. Peak pattern distributions in unstable mononucleotide repeat markers differed between samples with and without matching Penta-C and Penta-D loci (*p* < 0.01, Supplementary Table S2).

Peak patterns and instability rates in Penta-C and Penta-D were not influenced by the percentage of viable tumor cells. In this study, the average tumor content was comparable between MSS and MSI-H samples (65.2% vs. 69.8%). Samples with both markers matching, both markers non-matching, and only one marker matching contained 70.0%, 69.3%, and 72.8% viable tumor cells, respectively. The mean tumor content in samples showing a duplication in the longer and a deletion in the shorter of two pentanucleotide peaks was 71.0% and 67.7% in Penta-C and 66.5% and 70.7% in Penta-D, respectively.

Data on instability of the Penta-C and Penta-D pentanucleotide markers in CRC are very limited in the literature. To our knowledge, this is the first study to examine associations between mono- and pentanucleotide peak patterns and their correlations with clinical parameters in CRC. Only a few previous studies, each with small sample size, have evaluated instability in pentanucleotide markers. Murphy et al. (2006) [15] analyzed 34 samples but lacked detailed clinical data; none of the 11 MSI-H cases in that cohort exhibited instability in both pentanucleotides. Sample identification in MSI testing is based on size comparison in both pentanucleotide markers. The authors reported 17% allelic imbalance or loss of heterozygosity (LOH) in Penta-C and Penta-D among 23 MSS cases, with 5 bp deletions being the most common type of change. They also observed size shifts in 36% of Penta-C and 45% of Penta-D loci in MSI-H samples. Another small study [10] found instability in 34.5% (30/87) of Penta-D and 13.8% (12/87) of Penta-C loci in MSI-H CRC, but did not characterize peak patterns or assess associations of peak patterns with clinical parameters. To our knowledge, our study evaluating instability in Penta-C and Penta-D across 5218 samples from 2609 patients represents the largest and most exhaustive investigation of this kind to date.

We recently reported instability in pentanucleotide markers in EC [23]. In the present study, MSI analysis based on mononucleotide markers revealed lower MSI frequencies in CRC than in EC. The proportions of MSI-H, MSI-low, and MSS cases in CRC were 16.9%, 0.3%, and 82.8%, respectively, compared with 22.5%, 3.7%, and 73.8% in EC. The lower MSI rate in CRC relative to EC is consistent with previously published data. Wang et al. (2017) [30] reported MSI-H, MSI-low, and MSS frequencies of 14.8%, 0.3%, and 84.9% in CRC and 22.0%, 1.1%, and 76.9% in EC. A meta-analysis by Lorenzi et al. (2020) [31] found MSI in 13.0% of CRC and 26.0% of EC cases. Similarly, Kavun et al. (2023) [32] reported MSI frequencies of 10.2% in CRC and 21.9% in EC.

In the present study, pentanucleotide instability, when both Penta-C and Penta-D loci were considered together, was comparable in MSS samples (0.3% vs. 0.4%) but higher in MSI-H CRC than in MSI-H EC (12.3% vs. 8.2%). This difference may reflect tumor-specific factors or the larger sample size in the current study (5218 vs. 648 samples). Previous studies have shown that minimal fragment shifts are more common in MSI-H EC than in MSI-H CRC, potentially reducing diagnostic accuracy in EC [33]. Consistently, Dedeurwaerdere et al. (2021) [34] reported that molecular MSI analysis had lower sensitivity for detecting dMMR in EC than in CRC. Similarly, Boyarskikh et al. (2023) [35] observed lower MSI testing accuracy in EC compared with CRC using targeted next-generation sequencing.

The 12.3% instability rate in the MSI-H CRC samples observed in our study may have broad implications for clinical laboratory practice. Several findings from our analysis are particularly relevant. First, the presence of instability in both mono- and pentanucleotide markers in 12.3% of the MSI-H cases indicates that other STR loci may also be unstable and, therefore, unreliable for sample identification in this subset of tumors. Cases exhibiting mismatches in both Penta-C and Penta-D may require repeat testing using fresh samples, use of commercial sample identification kits, independent verification with unrelated loci, or SNP genotyping. However, many of these approaches may not be feasible for routine implementation in busy clinical laboratories. If samples fail additional identity verification, potential explanations such as sample swaps, contamination, or rare biological events should be considered. Alternatively, the samples may be referred to NGS-based testing which evaluates a large number of loci for both sample identification and MSI analysis. Pentanucleotide markers in MSI-H cases should be interpreted with additional caution due to the increased possibility of mismatches resulting from dMMR in these patients. Unfortunately, some diagnostic laboratories may interpret discordant pentanucleotide results as potential evidence of contamination or sample mix-ups and, consequently, choose not to report MSI findings in those cases.

Second, mono- and pentanucleotide peak patterns varied among MSI-H patients and correlated with several clinical parameters. *MLH1* promoter hypermethylation was significantly associated with instability in Penta-C (*p* = 0.03), and loss of MMR protein expression correlated with instability in both Penta-C and Penta-D (*p* < 0.01; Table 3).

Third, analysis of 4320 MSS tumor and matched normal tissue samples from 2160 patients in our study demonstrated that sample mix-ups are uncommon in cases without dMMR. Both pentanucleotide markers did not match in only 0.3% of the MSS samples. Sample misidentification remains a fundamental but under-studied problem in clinical molecular laboratories. It is estimated that specimen mix-ups occur in up to 3.5% of pathology samples [36]. In 2018, the Centers for Medicare & Medicaid Services (CMS) reported that 3.8% of the laboratory deficiencies identified during inspections of 9655 laboratories involved sample source errors. Sample swaps can lead to significant diagnostic errors with potentially serious consequences for patients.

The mechanisms responsible for the distinct peak patterns observed in unstable mono- and pentanucleotide repeat markers have been rarely explored. It has been hypothesized that these patterns arise from the accumulation of replication errors that occur during tumor progression [37]. Sequential slippage events during DNA replication are thought to cause progressive changes in allelic size, leading to shifts in fragment length over multiple cell divisions. As a result, the larger normal-specific alleles located on the right side of the electropherogram are gradually replaced by the smaller tumor-specific alleles on the left, producing an apparent right-to-left shift and generating the characteristic serrated or displaced peak patterns observed in MSI-H tumors.

A key limitation of our study was the small and, in some cases, imbalanced sample size across specific subcategories. For example, results related to patient ethnicity should be interpreted with caution due to unequal group representation. In Ohio, individuals identifying as White, Black, and Asian constitute approximately 90%, 8%, and 1% of the population, respectively. Our results may not fully reflect the diversity of minority groups although similar proportions were observed in our patient cohort. Additionally, small allelic shifts, complex peak profiles, and “bleed-through” peaks occur more frequently in mononucleotide than in pentanucleotide markers, contributing to a more subjective interpretation of mononucleotide electropherogram patterns. Sample numbers were also limited for several rare peak types in our study. We did not have sufficient data to support a multiple regression analysis. Therefore, our results may not have fully accounted for potential confounding factors such as anatomic site, tumor purity, or IHC category. Finally, this analysis did not assess loss of heterozygosity, copy number variation, or other complex genetic alterations, which may influence microsatellite stability.

In summary, our findings indicate that the Penta-C and Penta-D loci are unstable in 12.3% of the MSI-H samples. This instability is likely attributable to dMMR, as it correlates strongly with MSI status and loss of MMR protein expression by IHC. The concurrent instability of mono- and pentanucleotide markers in these MSI-H tumors suggests that other STR loci may also be unstable and, therefore, not reliable for sample identification in this subset of patients. These observations have practical implications for optimizing MSI testing protocols used to screen patients for LS and to identify patients who may benefit from cancer immunotherapy. Furthermore, peak pattern variability in unstable pentanucleotide markers appears to correlate with specific clinical characteristics. Future research into the mechanisms driving these distinct peak patterns may enhance our understanding of the molecular processes underlying tumorigenesis and MSI development.

## 5. Conclusions

This study demonstrated that the Penta-C and Penta-D pentanucleotide markers are unstable in 12.3% of MSI-H CRCs, primarily due to dMMR. Instability in these markers correlates with *MLH1* promoter hypermethylation and loss of MMR protein expression, whereas MSS samples show minimal instability, indicating extremely rare sample misidentification. The concurrent instability of mono- and pentanucleotide markers in MSI-H samples suggests that other short tandem repeat loci may also be unreliable for sample identification in MSI-H tumors. These findings highlight the need for supplemental identification markers to ensure accurate sample matching in clinical laboratories. Peak pattern variability appears to reflect tumor-specific replication errors and correlates with clinical parameters, offering insight into underlying mechanisms of genomic instability. These results should help in optimizing MSI testing protocols, improving LS screening, and identifying patients who may benefit from immunotherapy.

## Figures and Tables

**Table 1:** Clinical characteristics of the 2609 patients included in this study.

Parameter		*n*	%
Age younger than 50 yr ^a^		537	20.6
Race	White	2354	90.6
	Black	204	7.9
	Asian	26	1
	Others	13	0.5
Tissue ^b^	Colon	1947	74.6
	Rectum	602	23.1
	Liver	24	0.9
	Omentum	11	0.4
	Other	25	1
MSI status ^c^	MSI-H	440	16.9
	MSI-low	9	0.3
	MSS	2160	82.8
HYP ^d^	Present	259	83.5
	Absent	51	16.5
IHC ^e^	IHC intact	2158	82.9
	Absent MLH1/PMS2	322	12.4
	Absent MSH2/MSH6	49	1.9
	Absent PMS2	28	1.1
	Absent MSH6	20	0.8
	Others	26	1

^a^ Age < 50 years at the time of diagnosis. ^b^ Metastatic colorectal cancer samples were obtained from the liver, omentum, lymph node, abdominal mass biopsy, or other metastatic sites. ^c^ In total, 54 of these 440 cases (12.3%) had mismatches in both Penta-C and Penta-D (Table 2). ^d^ HYP = Rate of *MLH1* promoter hypermethylation among patients with loss of MLH1/PMS2 expression in immunohistochemistry. ^e^ IHC = Immunohistochemistry.

**Table 2:** MSI status and source of normal DNA in patients stratified based on Penta-C and Penta-D matching. ^a^ Same = normal DNA was isolated from tissue block(s) same as tumor. ^b^ Different = normal DNA was isolated from tissue block(s) different from tumor.

		Penta-C				Penta-D				Both Penta-C and Penta-D							
		Match		Does Not Match		Match		Does Not Match		Match		Do Not Match		Only Penta-C Matches		Only Penta-D Matches	
	*n*	*n*	%	*n*	%	*n*	%	*n*	%	*n*	%	*n*	%	*n*	%	*n*	%
MSI status																	
MSI-H	440	321	73	119	27	265	60.2	175	39.8	200	45.5	54	12.3	121	27.5	65	14.8
MSI-low	9	8	88.9	1	11.1	8	88.9	1	11.1	7	77.8	0	0	1	11.1	1	11.1
MSS	2160	2114	97.9	46	2.1	2117	98	43	2	2077	96.2	6	0.3	37	1.7	40	1.9
Source of normal DNA																	
Blood	376	359	95.5	17	4.5	341	90.7	35	9.3	332	88.3	8	2.1	27	7.2	9	2.4
Same ^a^	1412	1316	93.2	96	6.8	1297	91.9	115	8.1	1234	87.4	33	2.3	82	5.8	63	4.5
Different ^b^	510	467	91.6	43	8.4	463	90.8	47	9.2	437	85.7	17	3.3	30	5.9	26	5.1

**Table 3:** Clinical parameters in patients stratified by Penta-C and Penta-D matching. ^a^ Unstable marker = average unstable mononucleotide marker (out of five). ^b^ Age < 50 yr = age younger than 50 years at the time of diagnosis.

	Penta-C				Penta-D				Both Penta-C and Penta-D							
	Matches		Does Not Match		Matches		Does Not Match		Match		Do Not Match		Only Penta-C Matches		Only Penta-D Matches	
Clinical Parameters	*n*	%	*n*	%	*n*	%	*n*	%	*n*	%	*n*	%	*n*	%	*n*	%
Unstable marker ^a^	0.65		3.56		0.59		3.95		0.42		4.45		3.75		3.03	
*MLH1* hypermethylation																
Present	187	69	84	31	163	60.1	108	39.9	117	43.2	38	14	70	25.8	46	17.0
Absent	122	78.7	33	21.3	99	63.9	56	36.1	81	52.3	15	9.7	41	26.5	18	11.6
Age < 50 yr ^b^																
Yes	512	95.3	25	4.7	503	93.7	34	6.3	485	90.3	7	1.3	27	5	18	3.4
No	1927	93.2	140	6.8	1883	91.1	184	8.9	1795	86.8	52	2.5	132	6.4	88	4.3
IHC																
Absent MLH1/PMS2	223	69.3	99	30.7	191	59.3	131	40.7	136	42.2	44	13.7	87	27	55	17.1
Absent MSH2/MSH6	36	73.5	13	26.5	31	63.3	18	36.7	25	51	7	14.3	11	22.4	6	12.2
Absent PMS2	25	89.3	3	10.7	14	50	14	50	13	46.4	2	7.1	12	42.9	1	3.6
Absent MSH6	20	100	0	0	16	80	4	20	16	80	0	0	4	20	0	0
IHC intact	2111	97.8	47	2.2	2110	97.8	48	2.2	2069	95.9	6	0.3	42	1.9	41	1.9
Others	15	78.9	4	21.1	13	68.4	6	31.6	12	63.2	1	5.3	3	15.8	3	15.8
Race																
White	2201	93.5	153	6.5	2146	91.2	208	8.8	2049	87	56	2.4	152	6.5	97	4.1
Black	193	94.6	11	5.4	195	95.6	9	4.4	187	91.7	3	1.5	6	2.9	8	3.9
Asian	24	92.3	2	7.7	25	96.2	1	3.8	24	92.3	1	3.8	0	0	1	3.8
Other	13	100	0	0	13	100	0	0	13	100	0	0	0	0	0	0

**Table 4:** Peak types in unstable Penta-C and Penta-D. ^a^ 5 bp DEL = 5 bp deletion in a single peak; 5 bp DUP = 5 bp duplication in a single peak; 5 bp DEL short = 5 bp deletion in the shortest of two or more peaks; 5 bp DUP tall = 5 bp duplication in the tallest of two or more peaks. Representative electropherograms for these peak types are shown in Figure 1. ^b^ Indeterminate pattern: the alteration cannot be reliably determined to result from deletion(s), duplication(s), or both in the germline alleles. ^c^ Complex = multiple types of instability identified within the same sample.

	Penta-C		Penta-D	
Peak Types	*n*	%	*n*	%
5 bp DEL ^a^	29	18.4	24	11
5 bp DUP	8	5.1	8	3.7
5 bp DEL short	34	21.5	56	25.7
5 bp DUP tall	15	9.5	22	10.1
Indeterminate ^b^	48	30.4	72	33
Complex ^c^	24	15.2	36	16.5
Total	158	100	218	100

**Table 5:** Clinical characteristics of patients exhibiting different peak pattern types in unstable Penta-C and Penta-D loci. Representative electropherograms of these peak patterns are shown in Figure 1. ^a^ Unstable marker = average number of unstable mononucleotide repeat markers (out of five).

		5 bp DEL		5 bp DUP		5 bp DEL Short		5 bp DUP Tall		Indeterminate/ Complex	
Pentanucleotide Marker		*n*	%	*n*	%	*n*	%	*n*	%	*n*	%
Penta-C											
Unstable marker ^a^		4.77		2.38		4.47		3.21		4.17	
*MLH1* hypermethylation											
	Present	19	24.1	4	5.1	23	29.1	7	8.9	26	32.9
	Absent	8	25	1	3.1	8	25	3	9.4	12	37.5
Age < 50 yr at the time of diagnosis											
	Yes	3	15.8	1	5.3	5	26.3	3	15.8	7	36.8
	No	26	22.8	6	5.3	29	25.4	12	10.5	41	36.0
IHC											
	IHC intact	2	9.1	3	13.6	4	18.2	5	22.7	8	36.4
	Absent MLH1/PMS2	22	21.4	4	3.9	29	28.2	9	8.7	39	37.9
	Absent MSH2/MSH6	3	75	0	0	1	25	0	0	0	0
	Absent PMS2	1	33.3	1	33.3	0	0	1	33.3	0	0
	Absent MSH6	0	0	0	0	0	0	0	0	0	0
	Others	1	50	0	0	0	0	0	0	1	50
Penta-D											
Unstable marker		4.08		3.13		3.96		3.5		4.28	
*MLH1* hypermethylation											
	Present	13	14.9	4	4.6	24	27.6	10	11.5	36	41.4
	Absent	5	9.3	1	1.9	20	37	6	11.1	22	40.7
Age < 50 yr at the time of diagnosis											
	Yes	4	9.5	1	2.4	9	21.4	17	40.5	11	26.2
	No	20	14.4	7	5	46	33.1	5	3.6	61	43.9
IHC											
	IHC intact	4	10.8	3	8.1	12	32.4	6	16.2	12	32.4
	Absent MLH1/PMS2	14	13	5	4.6	30	27.8	12	11.1	47	43.5
	Absent MSH2/MSH6	1	5.9	0	0	6	35.3	2	11.8	8	47.1
	Absent PMS2	2	15.4	0	0	6	46.2	2	15.4	3	23.1
	Absent MSH6	1	33.3	0	0	2	66.7	0	0	0	0
	Others	2	50	0	0	0	0	0	0	2	50

## Data Availability

Data supporting the findings of this study are available from the corresponding author, Heather Hampel, upon reasonable request.

## Supplementary Materials

The following supporting information can be downloaded at: https://www.mdpi.com/article/10.3390/curroncol33040205/s1, Table S1: Total number of peaks in Penta-C and Penta-D; Table S2: Peak patterns in unstable mononucleotide repeat markers in patients with or without matching Penta-C and Penta-D. Mono = mononucleotide repeat marker, Pattern = peak patterns in mononucleotide repeat markers, Typical = two separate peaks with different size, Serrated = peaks with incremental changes in fragment height, Others = all peak types that are not “Typical” or “Serrated”. The “Others” category includes the “flat” (i.e., peaks with fragments with similar height located in the middle, resulting in a flat appearance), and peaks completely shifted to the left on an electropherogram due to the (nearly) total absence of the normal tissue-specific peaks; Table S3: Associations of peak patterns in unstable mononucleotide repeat markers with clinical parameters. Mono = mononucleotide repeat marker, Pattern = peak patterns in mononucleotide repeat markers, Marker = average unstable mononucleotide markers (out of five), Typical = two separate peaks with different size, Serrated = peaks with incremental changes in fragment height, Others = all peak types that are not “Typical” or “Serrated”. The “Others” category includes the “flat” (i.e., peaks with fragments with similar height located in the middle, resulting in a flat appearance), and peaks completely shifted to the left on an electropherogram due to the (nearly) total absence of the normal-specific peaks; Table S4: Associations of peak patterns in unstable mononucleotide repeat markers with race. Mono = mononucleotide repeat marker, Pattern = peak patterns in mononucleotide repeat markers, Typical = two separate peaks with different size, Serrated = peaks with incremental changes in fragment height, Others = all peak types that are not “Typical” or “Serrated”. The “Others” category includes the “flat” (i.e., peaks with fragments with similar height located in the middle, resulting in a flat appearance), and peaks completely shifted to the left on an electropherogram due to the (nearly) total absence of the normal-specific peaks.
