# Supplementary material for: Instability in Pentanucleotide Markers in a Subset of Microsatellite Instability-High Colorectal Cancer
Source: Curr Oncol. 2026 Apr 2;33(4):205. doi: 10.3390/curroncol33040205 (PMC13114762; doi:10.3390/curroncol33040205)
Supplement: Supplementary file 1 [file curroncol-33-00205-s001.zip › curroncol-4123664-supplementary.pdf]

Supplementary Materials

Supplementary Table S1. Total number of peaks in Penta-C and Penta-D.

			Pentanucleotide peak numbers							
			1 peak		2 peaks		3 peaks		≥4 peaks	
MSI	Pentanucleotide	*n*	*n*	%	*n*	%	*n*	%	*n*	%
MSI-H	Penta-D normal	440	99	22.5	340	77.3	1	0.2	0	0
	Penta-D tumor	440	65	14.8	229	52	122	27.7	24	5.5
	Penta-C normal	440	108	24.5	331	75.2	1	0.2	0	0
	Penta-C tumor	440	75	17	278	63.2	75	17	12	2.7
MSS	Penta-D normal	2,160	394	18.2	1763	81.6	3	0.1	0	0
	Penta-D tumor	2,160	388	18	1741	80.6	31	1.4	0	0
	Penta-C normal	2,160	550	25.5	1606	74.4	4	0.2	0	0
	Penta-C tumor	2,160	550	25.5	1592	73.7	18	0.8	0	0

Supplementary Table S2. Peak patterns in unstable mononucleotide repeat markers in patients with or without matching Penta-C and Penta-D. Mono = mononucleotide repeat marker, Pattern = peak patterns in mononucleotide repeat markers, Typical = two separate peaks with different size, Serrated = peaks with incremental changes in fragment height, Others = all peak types that are not "Typical" or "Serrated". The "Others" category includes the "flat" (i.e., peaks with fragments with similar height located in the middle, resulting in a flat appearance), and peaks completely shifted to the left on an electropherogram due to the (nearly) total absence of the normal tissue-specific peaks.

		Penta-C				Penta-D				Both Penta-C and Penta-D							
		Match		Does not match		Match		Does not match		Both match		Both do not match		Only Penta-C matches		Only Penta-D matches	
Mono	Pattern	*n*	%	*n*	%	*n*	%	*n*	%	*n*	%	*n*	%	*n*	%	*n*	%
NR-21																	
	Typical	243	69.2	108	30.8	201	57.3	150	42.7	140	39.9	47	13.4	103	29.3	61	17.4
	Serrated	30	85.7	5	14.3	25	71.4	10	28.6	23	65.7	3	8.6	7	20	2	5.7
	Others	28	82.4	6	17.6	22	64.7	12	35.3	20	58.8	4	11.8	8	23.5	2	5.9
NR-24																	
	Typical	217	69.3	96	30.7	175	55.9	138	44.1	122	39.0	43	13.7	95	30.4	53	16.9
	Serrated	44	80.0	11	20.0	36	65.5	19	34.5	30	54.5	5	9.1	14	25.5	6	10.9
	Others	27	79.4	7	20.6	25	73.5	9	26.5	21	61.8	3	8.8	6	17.6	4	11.8
BAT-25																	
	Typical	227	68.8	103	31.2	191	57.9	139	42.1	133	40.4	45	13.6	94	28.5	58	17.6
	Serrated	46	93.9	3	6.1	34	69.4	15	30.6	32	65.3	1	2	14	28.6	2	4.1
	Others	33	75.0	11	25.0	27	61.4	17	38.6	23	52.3	7	15.9	10	22.7	4	9.1
BAT-26																	
	Typical	241	70.9	99	29.1	195	57.4	145	42.6	141	41.5	45	13.2	100	29.4	54	15.9
	Serrated	18	81.8	4	18.2	17	77.3	5	22.7	15	68.2	2	9.1	3	13.6	2	9.1
	Others	39	75.0	13	25	34	65.4	18	34.6	27	51.9	6	11.5	12	23.1	7	13.5
MONO-27																	
	Typical	238	70.2	101	29.8	192	56.6	147	43.4	134	39.5	43	12.7	104	30.7	58	17.1
	Serrated	37	84.1	7	15.9	35	79.5	9	20.5	31	70.5	3	6.8	6	13.6	4	9.1
	Others	26	74.3	9	25.7	21	60	14	40	19	54.3	7	20	7	20	2	5.7

Supplementary Table S3. Associations of peak patterns in unstable mononucleotide repeat markers with clinical parameters. Mono = mononucleotide repeat marker, Pattern = peak patterns in mononucleotide repeat markers, Marker = average unstable mononucleotide markers (out of five), Typical = two separate peaks with different size, Serrated = peaks with incremental changes in fragment height, Others = all peak types that are not "Typical" or "Serrated". The "Others" category includes the "flat" (i.e., peaks with fragments with similar height located in the middle, resulting in a flat appearance), and peaks completely shifted to the left on an electropherogram due to the (nearly) total absence of the normal-specific peaks.

			Tissue source				Age at the time of diagnosis				*MLH1* hypermethylation			
			Colon		Rectum		< 50 yr		≥ 50 yr		Absent		Present	
Mono	Pattern	Marker	*n*	%	*n*	%	*n*	%	*n*	%	*n*	%	*n*	%
NR-21														
	Typical	4.92	329	94.3	20	5.7	54	15.4	296	84.6	110	32.9	224	67.1
	Serrated	4.27	31	88.6	4	11.4	7	20.6	27	79.4	9	27.3	24	72.7
	Others	4.8	28	84.8	5	15.2	8	23.5	26	76.5	20	60.6	13	39.4
NR-24														
	Typical	4.96	294	94.5	17	5.5	43	13.7	270	86.3	48	28.2	122	71.8
	Serrated	4.74	51	92.7	4	7.3	9	16.7	45	83.3	25	45.5	30	54.5
	Others	4.72	31	91.2	3	8.8	9	26.5	25	73.5	17	56.7	13	43.3
BAT-25														
	Typical	4.93	307	93.9	20	6.1	47	14.3	281	85.7	46	29.9	108	70.1
	Serrated	4.32	44	89.8	5	10.2	12	25	36	75.0	27	55.1	22	44.9
	Others	4.86	41	93.2	3	6.8	10	22.7	34	77.3	20	45.5	24	54.5
BAT-26														
	Typical	4.92	320	94.4	19	5.6	48	14.2	290	85.8	104	32	221	68
	Serrated	4.2	19	90.5	2	9.5	7	33.3	14	66.7	10	47.6	11	52.4
	Others	4.68	46	90.2	5	9.8	11	21.2	41	78.8	23	46	27	54
MONO-27														
	Typical	4.92	318	94.4	19	5.6	48	14.2	289	85.8	103	32.1	218	67.9
	Serrated	4.5	39	88.6	5	11.4	13	30.2	30	69.8	21	50	21	50
	Others	4.56	31	88.6	4	11.4	5	14.3	30	85.7	13	39.4	20	60.6

Supplementary Table S4. Associations of peak patterns in unstable mononucleotide repeat markers with race. Mono = mononucleotide repeat marker, Pattern = peak patterns in mononucleotide repeat markers, Typical = two separate peaks with different size, Serrated = peaks with incremental changes in fragment height, Others = all peak types that are not "Typical" or "Serrated". The "Others" category includes the "flat" (i.e., peaks with fragments with similar height located in the middle, resulting in a flat appearance), and peaks completely shifted to the left on an electropherogram due to the (nearly) total absence of the normal-specific peaks.

Mono	Pattern	White	% White	Black	% Black	Asian	% Asian	Other	% Other
NR-21									
	Typical	329	93.7	16	4.6	3	0.9	3	0.9
	Serrated	31	88.6	3	8.6	0	0	1	2.9
	Others	31	91.2	1	2.9	1	2.9	1	2.9
NR-24									
	Typical	293	93.9	14	4.5	3	1	2	0.6
	Serrated	52	94.5	3	5.5	0	0	0	0
	Others	28	82.4	2	5.9	2	5.9	2	5.9
BAT-25									
	Typical	312	94.8	13	4	3	0.9	1	0.3
	Serrated	40	83.3	4	8.3	3	6.3	1	2.1
	Others	39	88.6	3	6.8	0	0	2	4.5
BAT-26									
	Typical	320	95	13	3.9	3	0.9	1	0.3
	Serrated	20	90.9	2	9.1	0	0	0	0
	Others	44	84.6	4	7.7	2	3.8	2	3.8
MONO-27									
	Typical	318	94.4	14	4.2	4	1.2	1	0.3
	Serrated	38	86.4	4	9.1	1	2.3	1	2.3
	Others	31	88.6	3	8.6	0	0.0	1	2.9
